# Where did the finch go? Insights from radio telemetry of the medium ground finch (*Geospiza fortis*)

**DOI:** 10.1002/ece3.8768

**Published:** 2022-04-26

**Authors:** Marc‐Olivier Beausoleil, Carlos Camacho, Julio Rabadán‐González, Kristen Lalla, Roxanne Richard, Paola Carrion‐Avilés, Andrew P. Hendry, Rowan D. H. Barrett

**Affiliations:** ^1^ 5620 Redpath Museum and Department of Biology McGill University Montréal QC Canada; ^2^ Department of Biological Conservation and Ecosystem Restoration Instituto Pirenaico de Ecología—CSIC Jaca Spain; ^3^ 5193 Department of Biology Centre for Animal Movement Research (CAnMove) Lund University Lund Sweden; ^4^ Observation.org Spain Seville Spain; ^5^ 5620 Department of Natural Resource Sciences McGill University Sainte‐Anne‐de‐Bellevue QC Canada

**Keywords:** behavior, communal roosting, *Geospiza fortis*, habitat selection, home range, spatial ecology

## Abstract

Movement patterns and habitat selection of animals have important implications for ecology and evolution. Darwin's finches are a classic model system for ecological and evolutionary studies, yet their spatial ecology remains poorly studied. We tagged and radio‐tracked five (three females, two males) medium ground finches (*Geospiza fortis*) to examine the feasibility of telemetry for understanding their movement and habitat use. Based on 143 locations collected during a 3‐week period, we analyzed for the first time home‐range size and habitat selection patterns of finches at El Garrapatero, an arid coastal ecosystem on Santa Cruz Island (Galápagos). The average 95% home range and 50% core area for *G*.* fortis* in the breeding season was 20.54 ha ± 4.04 ha *SE* and 4.03 ha ± 1.11 ha *SE*, respectively. For most of the finches, their home range covered a diverse set of habitats. Three finches positively selected the dry‐forest habitat, while the other habitats seemed to be either negatively selected or simply neglected by the finches. In addition, we noted a communal roosting behavior in an area close to the ocean, where the vegetation is greener and denser than the more inland dry‐forest vegetation. We show that telemetry on Darwin's finches provides valuable data to understand the movement ecology of the species. Based on our results, we propose a series of questions about the ecology and evolution of Darwin's finches that can be addressed using telemetry.

## Graphical abstract

Movement patterns and habitat selection of animals have important implications for ecology and evolution. Darwin's finches are a classic model system for ecological and evolutionary studies, yet their spatial ecology remains poorly studied due to concerns about the use of telemetry. In this paper, we test the feasibility of radio‐tracking medium ground finches and demonstrate its utility by characterizing for the first time the habitat selection patterns of breeding ground finches in the arid coastal zone of Santa Cruz Island (Galápagos).

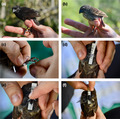

## 1 INTRODUCTION

The way in which animals move across the landscape has important implications for ecology and evolution: migration influences nutrient transfers, dispersal influences speciation, habitat choice influences natural selection, and home ranges influence competition (Holyoak et al., 2008; Jeltsch et al., 2013; Nathan, 2008). Hence, our knowledge of any model system in ecology and evolution benefits critically from an understanding of how an organism moves across its landscape. Darwin's finches on the Galápagos Islands are a classic system in evolutionary ecology (Grant, 1999; Grant & Grant, 2014), with a long history of research on morphological variation (Grant, 1999; Lack, 1947), growth and development (Grant, 1981), diet (De León et al., 2014), mate choice and species recognition (Grant & Grant, 1997; Podos, 2001), genomics (Chaves et al., 2016; Enbody et al., 2021; Lamichhaney et al., 2015, 2016, 2018), and habitat use (Grant, 1999; Grant & Grant, 2014). However, their movement ecology is poorly understood. At a small scale, direct observations and capture–recapture studies have shed some light on their natal and breeding dispersal (Grant, 1999), and breeding territory size (Boag & Grant, 1984; Grant & Grant, 1989; Price, 1984). At a larger scale, genetic studies have revealed that migratory movement is limited, but not absent, between islands but high within islands (De León et al., 2010; Lamichhaney et al., 2018; Lawson et al., 2019; Petren et al., 2005). Yet there is a knowledge gap between the small‐ and large‐scale movement studies, especially for finches’ daily movement routines, home range (Burt, 1943), and core area size (loosely defined as a smaller portion of the home range). For example, our knowledge of the movement of finches across the landscape, including permanent (dispersal) and intermittent (normal activities of food gathering, mating, and caring for young) displacements, is limited. Furthermore, although some information is available on breeding territory size (Boag & Grant, 1984) and flocking behavior of nonbreeding ground finches during the dry season (Schluter, 1982; Swash & Still, 2005) and on dispersal of captive‐reared mangrove finches (*Camarhynchus heliobates*, Cunninghame et al., 2017), almost no information exists on habitat use or patterns of commuting behavior in Darwin's finches. Therefore, scientists and conservation biologists lack basic information about the habitat selection patterns of the finches, their daily movement routines across the landscape, and the intrinsic and extrinsic factors influencing such movements.

The Island of Santa Cruz encompasses diverse habitats that provide numerous opportunities for finches to select particular environments (Grant, 1999; Reeder & Riechert, 1975). But, determining the movement of birds on a large territory comes with a logistical challenge: the difficulty of tracking individual finches. Darwin's finches can be challenging to recapture/resight since they can move long distances and aggregate in wandering flocks after the breeding season or when dry conditions preclude breeding (Schluter, 1982; Swash & Still, 2005). In addition, the large population sizes occupying a broad territory compared to the limited number of banding sites, and the fact that some individuals with larger beaks are able to remove their bands, makes it a challenge to track individuals by standard mark–recapture methods. Furthermore, GPS tags are generally still too heavy for finches due to their small body size (body mass 22 g ± 6 g [average ± 2x *SD*]). Telemetry methods (e.g., radio‐tracking) might provide direct information on the movement and behavior of individual finches and radio tags are small enough to be deployed on finches, yet they have not been extensively used in the Galápagos (exceptions include Fessl et al., 2010 and Cunninghame et al., 2017). Concerns about the use of telemetry generally stem from the perception that data collection will be challenging due to features of the landscape (e.g., dense vegetation, inaccessible areas due to the volcanic structure of the landscape).

Despite these concerns, telemetry in general, and Very High Frequency (VHF) radio telemetry in particular, has been used to investigate movement patterns in small birds (Kenward, 2001; White & Garrott, 1990), thus informing habitat selection (Camacho et al., 2014), foraging range and roosting (Ginter & Desmond, 2005), postfledging dispersal (Fisher & Davis, 2011), and migration (Bégin‐Marchand et al., 2021). This approach has also been used on rare occasions in Darwin's finches in the Galápagos, primarily for conservation purposes. Miniature radio‐transmitters have been previously deployed on the woodpecker finch (*Camarhynchus pallidus)* (Cunninghame et al., 2017; Fessl et al., 2010), and also on the critically endangered mangrove finch (*C*. *heliobates*) to track the movement of captive‐reared juveniles (Cunninghame et al., 2013, 2015, 2017). However, the utility of these methods for eco‐evolutionary studies of Darwin's finches captured and released in the wild is unknown. Thus, we here explore the extent to which radio‐transmitter tagging methods are effective in this context.

Our aims are threefold: (a) Explore Darwin's finch movement and space use associated with different behaviors (e.g., diurnal activity, nesting, and roosting); (b) Ascertain data quantity and quality to determine what kind of insights can be gained in a 3‐week data collection period (the duration of battery life of the miniature radio‐transmitters); and (c) Identify the limitations of using radio telemetry methods given the topography of the volcanic terrain. To fulfil these aims, we deployed VHF radio telemetry tags on a focal sample of five medium ground finches (*Geospiza fortis*) on Santa Cruz in the Galápagos, Ecuador. We then estimated the home range and core area of these birds in the arid coastal zone and characterized their habitat selection patterns and movement behavior. Finally, we discuss the potential utility of these methods for addressing three key unresolved questions, which we believe would advance our understanding about the behavior, ecology, evolution, and conservation of Darwin's finches: (a) What ecological factors influence finch's home range size and location?; (b) How does finch movement impact their ecological interactions with other taxa?; and (c) What factors influence roosting behavior in finches?

## 2 METHODS

### 2.1 Capture and transmitter deployment

Our study took place at El Garrapatero, Santa Cruz Island, Galápagos, Ecuador (0°41′22.9″ S, 90°13′19.7″ W) from 22 February to 13 March 2019 (20 days), during the breeding season of Darwin's finches. This population has been studied since 2003, with systematic data on behavior, feeding ecology, and morphology collected on an annual basis (Beausoleil et al., 2019; De León et al., 2011; Hendry et al., 2009; Knutie et al., 2019; Podos, 2007). Our test sample consisted of five medium ground finches (*Geospiza fortis*)—three females and two males (Table 1)—captured at the same dry forest sites we use during our long‐term systematic mist netting operations at El Garrapatero (Beausoleil et al., 2019; De León et al., 2014; Hendry et al., 2009). Only actively breeding individuals (i.e., adult females showing either an active or regressing brood patch, and adult males showing a cloacal protuberance (Pyle, 1997)) were tagged to reduce the variability in home range differences (Pagen et al., 2000; Streby et al., 2011). We determined sex based on plumage coloration (Grant, 1999; Price, 1984).

**TABLE 1:** Banding data for each *Geospiza fortis* captured

Date^a^	Band	Frequency (MHz)	Sex^d^	Breeding stage	Tarsus (mm)	Mass (g)	Wing chord (mm)
2019–02–26	JP4645^b^	294	f	Laying eggs	21.92	21.6	69
2019–02–26	KGSK2033^c^	191	f	Feeding young	20.71	19.7	68
2019–02–21	LF0216	154	m	Building nest	22.02	19.7	70
2019–02–26	LF1233	059	m	Building nest	22.31	26.1	79
2019–02–26	LF1234	206	f	Incubating	21.20	23.1	68

^a^ Date when the finch was banded and/or a radio transmitter was deployed.

^b^ Recaptured bird, first banded in 2013.

^c^ Recaptured bird, first banded in 2016.

^d^ m: male, f: female.

**FIGURE A1:**
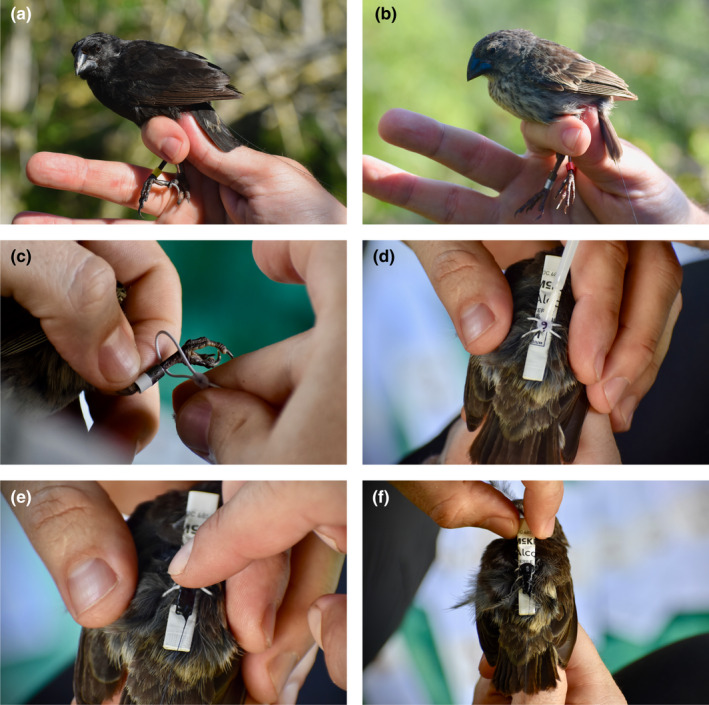
Attached radio transmitters on *Geospiza fortis* band number (a) LF1233 and (b) LF1234. Mounting process of the radio transmitter backpack on individual JP4645 (c‐d‐e‐f)

Each individual was fitted with a 0.56 g PicoPip Ag376 VHF radio transmitter (pulse length: 30 ms, pulse rate: 60 ppm, for about 3 weeks of battery life; Biotrack Ltd. UK). To attach the radio transmitter, we used a custom‐made leg‐loop harness made of a thin elastic band glued (cyanoacrylate) to the transmitter with a biodegradable paper in between to allow the harness to detach itself after 2–3 months (Naef‐Daenzer, 2007). We fitted the harness around the bird's legs and placed the transmitters on the bird's back (synsacrum) as described in Rappole and Tipton (1991; Figure A1) and cut the antenna to a final length of 11 cm to allow birds to move freely and avoid risks of entanglement (Dougill et al., 2000). The radio transmitter and harness represented <3% of the body mass of each individual (Murray & Fuller, 2000). For each bird, we measured tarsus length to the nearest 0.01 mm as an index of structural size (Senar & Pascual, 1997) and body mass to the nearest 0.1 g using a digital balance to adjust the size of the harness on which the VHF tag is attached. Individuals were banded with numbered Monel metal bands and a unique combination of plastic color bands for ease of identification in the field. Birds were released immediately after being measured and equipped with transmitters. The time from capture and tagging to release did not exceed 15 min.

### 2.2 Bird tracking

**FIGURE A2:**
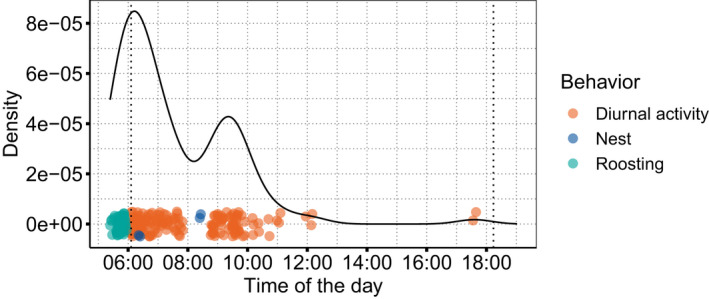
Density of sampling effort across time of the day at El Garrapatero. The points at the bottom were y‐jittered to better see the amount of sampling at a particular time of the day. The sunrise (0606 h) and sunset (1814 h) on the first day of March 2019 is shown as the dotted vertical lines

Tracking sessions began 24 h after tagging to facilitate resumption of normal behavior and activity, as confirmed by relocation and direct observation of tagged birds. Two observers simultaneously tracked radio‐tagged individuals for 3‐ to 5‐h sessions, usually in the morning between 0600 h and 1100 h, when birds are most active. They were also tracked opportunistically earlier (between 0500 h and 0600 h, before detecting any visual [e.g., flying silhouettes against the sky] or acoustic [e.g., dawn chorus] sign of bird activity) and later in the day (1700 h and 1800 h, after bird activity ceased in the evening; Figure A2) in order to locate the roosting sites. Each observer used a 3‐element antenna connected either to an ICOM IC‐R20 (Icom Inc., JP) or a SIKA (Biotrack Ltd., UK) portable receiver to record signal strength and direction. Sometimes the birds could be located and directly observed by tracking the VHF signal to its source (the nest or its immediate surroundings), and so their precise location was recorded using a Samsung A3 and J7 Pro phones with a Memento Database program (MementoDB Inc., mementodatabase.com) and ObsMapp (observation.org/apps/obsmapp/). Most often, to estimate their position we used bi‐triangulation of fixes based on an azimuthal telemetry model within the R‐package *razimuth* (50,000 iterations with 5,000 burn‐in; 600 prior due to detection range of antenna in the field; version 0.1.0; Gerber et al., 2018; R Core Team, 2021; R version 4.0.3). Directional bearings were estimated from accessible sites along the main road and the path to the beach. Bearings at angles around 90° to each other were generally preferred to obtain accurate estimates (mean of biangulation points =79.7° ± 3.1°*SE*, *N* = 90). Bearings that resulted in clearly erroneous estimates (e.g., those over the sea) were also removed from the dataset prior to analyses. Unusable locations represented 23% of the initial dataset (*N* = 286 fixes), and so the final sample size included 219 fixes acquired with telemetry (Table 2). We recorded additional fixes only after >20 min to minimize sample clustering. The birds were relocated sequentially at regular intervals to minimize bias in relocation effort. The average time between consecutive relocations on the same day was 2.31 h (range 1.67–2.78 h, *SE* =0.19 h, *N* = 5).

**TABLE 2:** Tracking parameters with home range and core area size estimates

Band	# tracking days	Duration of tracking period (days)^a^	No. fixes^b^	Number of points^c^	Home range size 95% (ha)	Core area size 50% (ha)	MCP^e^ 100% (ha)	*h*ref smoothing^f^
JP4645	10	13	37	30	7.58	1.17	3.37	46.27
KGSK2033	10	15	41	23	29.09	6.37	17.25	76.54
LF0216	9	14	39	31	14.86	1.90	8.83	55.16
LF1233	7	8	48	29	24.71	4.13	9.50	67.41
LF1234	8	12	54	30	26.47	6.60	11.84	64.57
			Total 219	143	20.54 ± 4.04^d^	4.03 ± 1.11^d^	10.16 ± 2.25^d^	

^a^ Number of days between the first and last tracking session (tracking span).

^b^ Number or bearings taken to bi‐triangulate the position of the finches.

^c^ Including direct observations, 2019 capture location, and the location estimated from the fixes.

^d^ Mean ± standard error (*SE*).

^e^ Minimum convex polygon (MCP) estimation of the home range.

^f^ Reference bandwidth, method of estimation of the smoothing parameter.

Observers also recorded the location of bird nests (when possible) and the tagged birds’ activity, either diurnal activity or roosting. Observers either triangulated nests (*N* = 1 bird) or found them (*N* = 3 birds) by estimating the approximate location of tagged birds and then moving closer using the signal strength until the nest was found and the identity of the bird was confirmed through their color band combinations. For one bird, the nest could not be located directly due to its limited accessibility (but see Figure A9). The location of roosts was estimated for all birds by biangulation during the night (Figure A2).

Direct observation of the behavior of the tagged individuals within the first 2–3 days after tagging enabled us to confirm their nesting status. The duration and periodicity of behavioral observations differed among individuals depending on the time needed to confirm their nesting status. Males collecting material to build their nests left the nest and returned back at short (1–5 min) regular intervals. Females incubating eggs or brooding chicks tended to remain in the nest for periods of at least 45 min (see Austin et al., 2019 for comparisons with other birds). Thus, the total time of observation per bird was ≤30 min in males (one single session) and 90–180 min in females (60‐min sessions on two to three consecutive days). This information also enabled us to link movement patterns, as determined from the radio tracking data, to the breeding stage of each bird and, therefore, to infer changes in the bird's breeding status throughout the study period. For instance, females that remained stationary (i.e., no apparent change in the signal strength or direction regardless of the tracking position) for 45 min or longer were assumed to continue incubation or nursing tasks. In contrast, rapid periodic changes in the strength of the signal from the location of the nest was taken as an indication of continued building activity in males or offspring provisioning in females (Orr, 1945; Price et al., 1983).

Prior to radio transmitter attachment on the birds, we estimated relocation error under field conditions by placing the VHF tags in random locations around the capture site and letting “blinded” observers find their position by taking bearing measurements. Then, as a measure of the error, we calculated the mean Euclidean distance between the estimated locations (using the razimuth package, see above) and the actual (georeferenced) location of the VHF tags (Figure A7).

### 2.3 Roost count

The tagged finches used a communal roosting area located outside the nesting area (except incubating females; see “Results”). We detected the communal roosting area by locating the birds 1 h before dawn (0500 h–0600 h). During this time, we considered a bird roosting if there was no apparent change in its signal strength or direction regardless of the tracking position. Given that one of our goals was to determine space use by the finches, we gathered data on the number of other (nontagged) finches using the communal roosting area. Once the location of roosting sites had been identified, two observers conducted a direct count of birds entering the roost during the evening (Table A1; Video 1). The site of the main roost was adjacent to the ocean, so the observers stood back‐to‐back perpendicularly to the shoreline to monitor all potential entrances (observer location 0°41′36.56″S, 90°13′18.16″W). These counts began near sunset (1800 h), before any bird was seen around the roosting area. The observers counted any finch or group of finches entering the roost and subtracted the (small) number of finches exiting the roost area (see Table A1). To avoid double‐counting, every observer informed their partner about birds passing from one visual range to another and flying out of the roost. Medium ground finches are the most abundant species in the study area (Beausoleil et al., 2019). However, during the census, we counted all finch species together because it is difficult to distinguish between Darwin's finch species from a distance due to similar plumage and size, especially under poor lighting conditions. The count lasted approximately 1 h, until finches stopped entering the roost.

**VIDEO 1:** Finches coming back to their roosting side 9 March 2019. The images were taken at about 18h10 near El Garrapatero's beach (0°41′38.54″S, 90°13′16.53″W). The video quality doesn't allow a proper finch count, but at least 50 finches were observed in about 5 min. (See https://ebird.org/hotspot/L3064040; Lalla, 2019)

### 2.4 Home range, core area, and habitat selection analyses

**FIGURE A3:**
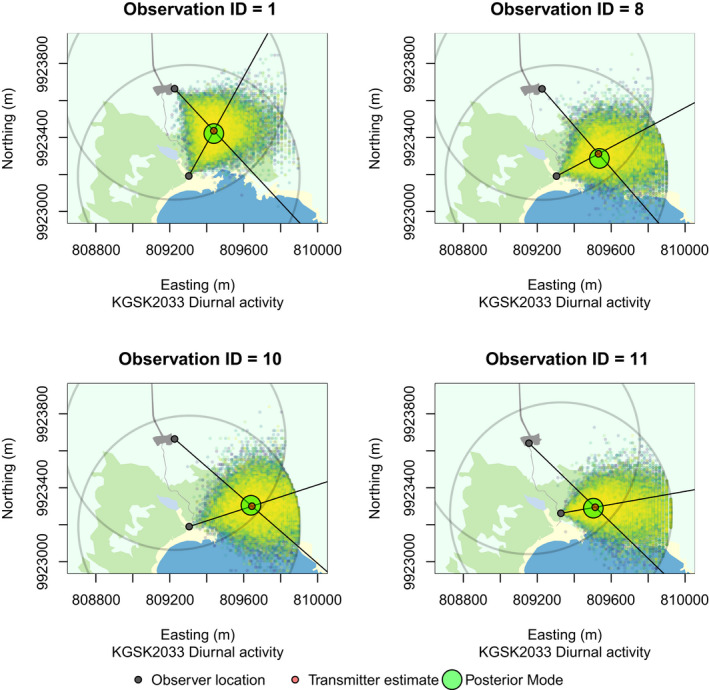
Bearing estimation from razimuth package (shown for only 4 points of individual KGSK2033 as an example; Gerber et al., 2018). The points behind the transmitter estimate and posterior mode are the MCMC iterations (50,000)

**FIGURE A4:**
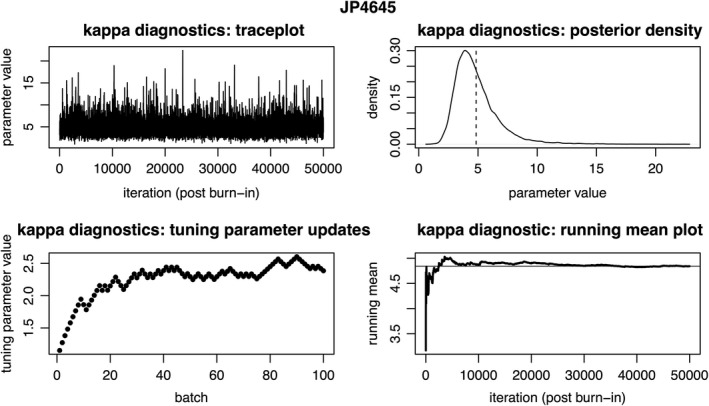
Diagnostic plot for razimuth model outputs for the individual JP4645

**FIGURE A5:**
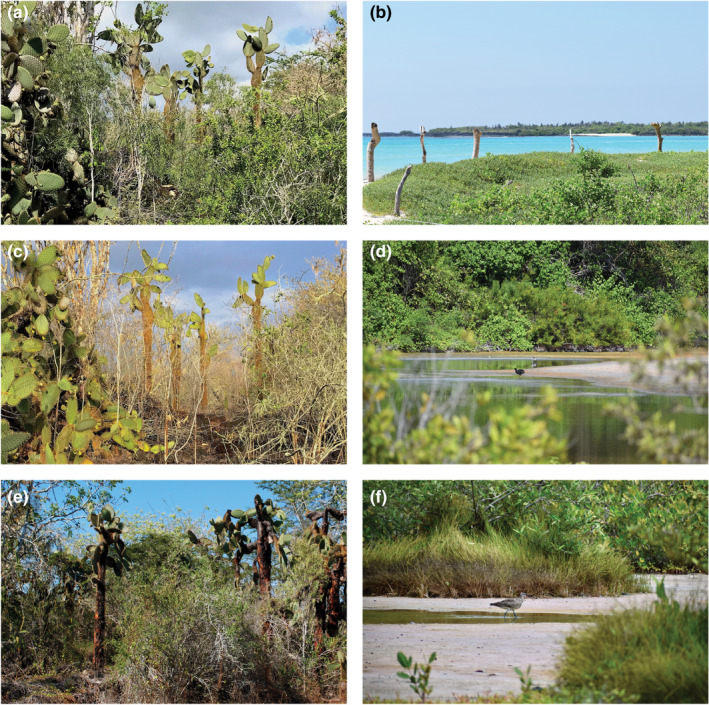
Differences between habitats at El Garrapatero. The arid natural zone of El Garrapatero (dry‐forest, a‐c‐e) and the beach transitional zone more utilized by tourists (b‐d‐f). The photo (b) was taken at the beach, and (d‐f) were taken at the site referred to as “inland water” which was considered the edge of the manzanillo forest. The same site can be seen at El Garrapatero (a and c), but (a) is in a wetter season in 2019 than (c) in 2018

For home range and habitat selection analyses, we combined the data from different sources (i.e., VHF‐inferred fixes, direct observation, and location of capture), after transformation to UTM coordinates. The azimuthal telemetry model (ATM) traceplot was visually inspected to ensure proper mixing of the Markov chain Monte Carlo (MCMC) chain for the concentration parameter *κ* (which controls the uncertainty in the ATM; see Gerber et al., 2018; Figures A3 and A4). The minimum number of points needed for accurate home range estimation was determined for each bird from the plateau of the rarefaction curve of minimum convex polygons (MCP 100%). To estimate home range size and core area, we used a bivariate normal kernel function using “*kernelUD*” (Utilization distribution) at 95% and 50%, respectively, from the adehabitatHR package (version 0.4.19; Calenge, 2006). For the smoothing parameter (*h*) for kernel estimation, we used the reference bandwidth (*h*ref) and constrained the area to be terrestrial (i.e., excluding the ocean). We used the sf library (version 0.9.8; Pebesma, 2018) to intersect the home ranges with the habitat types—as detailed below—and calculated the proportion of bird locations within habitat types and range overlap of habitat types for each finch. We mapped our results using ggplot (version 3.3.3; Wickham, 2016) and used satellite images and field observations for validation (Figure A5) to make our own habitat‐type polygons in QGIS (QGIS Development Team, 2021, version 3.16; Google Earth Pro, 2021). We categorized habitat types used by the finches as “beach,” “inland water” (a pond that can temporarily dry out), “Manzanillo forest” (coastal zone dominated by the tree *Hippomane mancinella* (poison apple) and other trees), “dry‐forest” dominated by *Opuntia echios* (prickly pear cactus) and *Bursera graveolens* (incense tree), and “paved road” encompassing a parking lot and road.

For the habitat selection analysis, we calculated the proportion of each habitat type within each home range (i.e., availability) and tested whether birds spent more or less time (the number of relocations) in each habitat than would be expected from its availability. Specifically, for each finch we compared the expected number of relocations in a habitat to the number of observed locations within that habitat with a chi‐square test. We calculated the Bonferroni corrected 95% confidence interval (from the proportion of observed locations of the bird in certain habitats to the total number of observations for that bird) as in Neu et al. (1974) and the direction of habitat selection (negative, neutral, or positive) as in Sierro et al. (2001). Specifically, if the observed area calculated as a proportion of a given habitat type in the home range was smaller than the lower bound of the Bonferroni confidence limit based on the proportion of bird locations in a particular habitat type, the bird was assumed to positively select the habitat. In the case where the proportion was greater than the upper confidence limit, it was considered to negatively select that habitat. In the case where the value lies inside the confidence interval, the bird was “neutral” with respect to that habitat. As a quantitative preference value for habitat selection, we also calculated the Jacobs’ index, in which a value of zero indicates a random utilization of the habitats, whereas a positive or negative value indicates a positive or negative selection of a habitat type, respectively (Jacobs, 1974; Lechowicz, 1982). Jacobs’ index has been used in other habitat selection studies (Revilla et al., 2000) and, contrary to the selection ratio, it is independent of the relative abundance of each habitat available to the birds (Jacobs, 1974). To determine diurnal and nocturnal differences in commuting behavior, we calculated the average distance (mean of all distances of the located finch to their nest) that the finches traveled from their nest during the day or at night.

## 3 RESULTS

Our sample of medium ground finches included three females at different stages of the breeding cycle and two nest‐building males (Table 1). The two males continued building nests throughout most of the tracking period, and one female (JP4645, Table A2) completed its clutch and then initiated incubation. The other females were already incubating (LF1234) or feeding offspring (KGSK2033) at the time of tagging. The resighted birds (all but LF1234) showed no sign of the negative impact of the radiotags on their diurnal behaviors. The tags remained in their original position until the end of the study in all but one bird: male LF0126 removed its tag after 2 weeks. The tag antenna was found bent, which may indicate that the bird was able to remove it with the beak once inside the nest.

**FIGURE A6:**
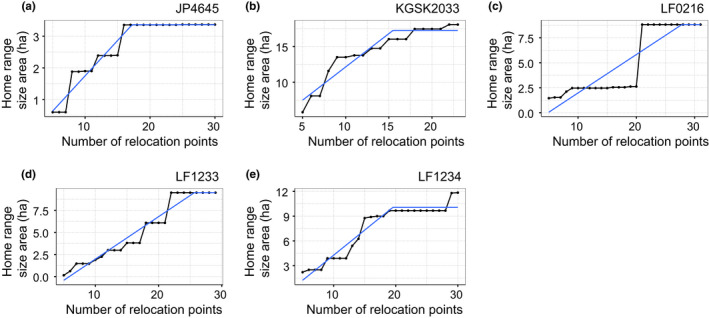
Minimum convex polygon rarefaction curve for each finch (a‐e). We used the function *mcp* (100%) from the adehabitatHR package to calculate the polygons (Calenge, 2006). All the points are ordered based on their sampling date. The blue line represents a Nonlinear Least Squares calculated with the function *nlsLM* from the minpack.lm package (Elzhov et al., 2016)

**FIGURE A7:**
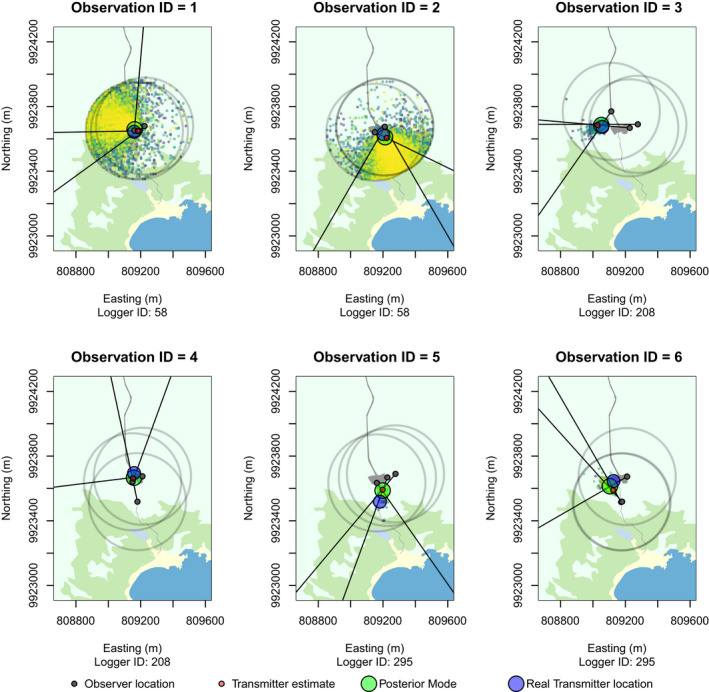
Bearing estimation from *razimuth* package (Gerber et al., 2018) quantifying the error location of the VHF emitters. The points not shown in the legend (the colour scale from yellow to purple) are the MCMC iterations (50,000)

We collected a total of 143 locations with a mean number of 28.6 locations per bird (range: 23–31; Video 1, Table 2). Of these locations, 81.1% (116 points) were estimated with the azimuthal telemetry model, 15.4% (22 points) through direct observation, and 3.5% (5 points) from mist‐netting (capture locations). The minimum number of fixes required for accurate home range estimation ranged from 17 to 28 locations depending on the bird (Figure A6). The mean relocation error was 30.11 m ± 8.98 m *SE* (range 13.13–70.93 m, *N* = 6). The total number of finches observed entering the communal roosting area in one evening was 669 finches (Table A1).

**FIGURE 1:**
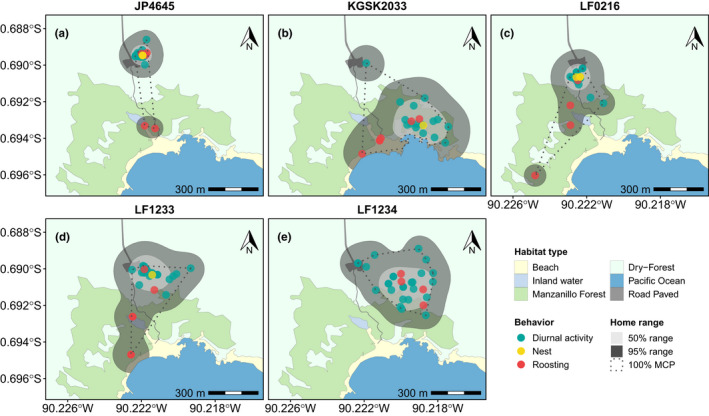
Maps of home ranges for radio‐tagged medium ground finches (a–e) at El Garrapatero on Santa Cruz Island, Galápagos. Each point represents the location of a finch

**FIGURE A8:**
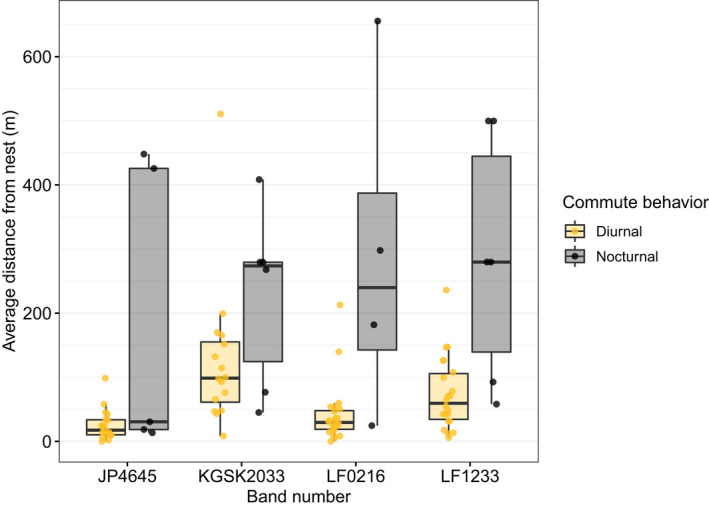
Average distances traveled by finches from their nest during the diurnal and nocturnal activities

**FIGURE A9:**
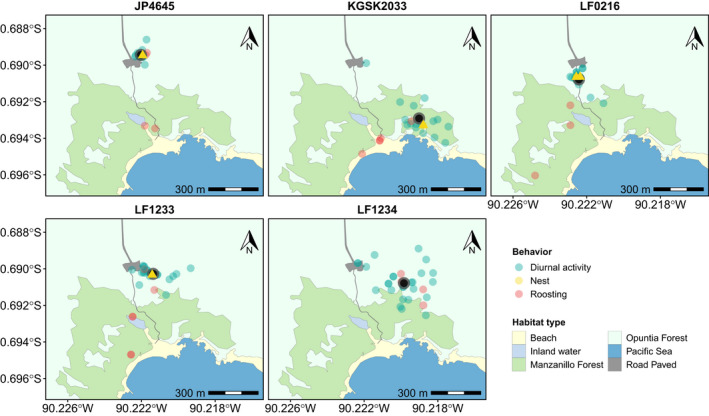
Estimation of nest location with the centroid of all diurnal activity locations of each finch. The yellow triangle is the known location of the nest of a bird. The black dot represents the centroid of all diurnal activity locations and the area around it is a buffer of 36.6 m determined by the upper limit of the 95% confidence interval of the distance between the nest and the centroid of all diurnal activity locations. Note that for female JP4645, the roosting points at the bottom of the map are from 2019‐02‐28 and 2019‐03‐01, before incubation started. From then on, this female remained on the nesting territory at night, as indicated by the three additional roosting points recorded during the incubation period, on 2019‐03‐04, 05, and 13. For the incubating female LF1234, the estimated location of the nest and that of nocturnal roosts are relatively close to each other, suggesting that this female also did not abandon the nesting territory during the night

The mean home range size (kernel 95%) was 20.54 ha ± 4.04 ha *SE* (range 7.58–29.09 ha, *N* = 5, Table 2) and the mean core area (kernel 50%) was 4.03 ha ± 1.11 ha *SE* (range 1.17–6.60 ha, *N* = 5). The tagged finches overlapped in their core areas, from 61% (76% for home range) from LF1233 on JP4645 and 43% (77% for home range) from LF1233 on LF0216 to <30% overlap in the other core areas (Figure 1, Table A3). The finches moved a greater distance (3.7 times more) on average from their nests to the roosting area (247 m ± 25 m *SE*, *N* = 4) compared to the distance they traveled during their daily activity (67 m ± 22 m *SE*, *N* = 4; Figure A8). The average daily commute distance (regardless of whether it is during day or night) was 102 m ± 21 m *SE*. Female JP4645 traveled to the communal roosting area at night during the egg‐laying stage, but remained on the nesting territory during the incubation stage (Figure A9). The incubating female LF1234 also remained on the putative nesting territory during the night (Figure A9), suggesting that the use of the communal roost is contingent upon the nesting status.

**FIGURE 2:**
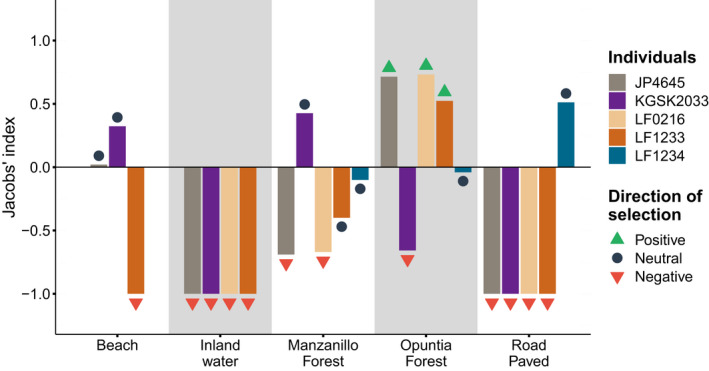
Jacobs’ index showing direction of selection for each habitat type for each finch. The gray shading is only for distinguishing the habitat types

Overall, the highest proportions of habitat types observed in the finches’ home ranges were arid zone dry‐forest (55.20%) and coastal zone “Manzanillo forest” (35.54%) (Table A4). Three finches (JP4645, LF0216, and LF1233) showed a positive selection for the dry‐forest, whereas one finch (KGSK2033) used this habitat less than expected by chance (Figure 2, Table 3 and Table A4). The rest of the habitat types were either negatively selected or not selected (Figure 2, Table 3). However, for only one male (LF0216), the use of a particular habitat (dry‐forest) deviated significantly from random expectation in a positive direction (*χ* ^2^ = 16.21, *p* = .001, *df* = 3; Figure 2, Table 3 and Table A4).

**TABLE 3:** Habitat selection analysis parameters

Band	Habitat type	Proportion of habitat in home range	Relocation number in habitat	Expected number of relocations	*χ* ^2^	*p*	Proportion of observed locations	95% CI	Direction of selection	Jacobs’ index
JP4645	Beach	0.032	1	0.96	0.001	.072	0.033	0–0.118	Neutral	0.02
	Inland water	0.025	0	0.76	0.759		0.000	0–0	Negative	−1
	Manzanillo forest	0.158	1	4.75	2.957		0.033	0–0.118	Negative	−0.69
	Dry‐forest	0.700	28	21.00	2.333		0.933	0.816–1.051	Positive	0.71
	Road paved	0.084	0	2.53	2.532		0.000	0–0	Negative	−1
	Total	1	30	30	8.58					
KGSK2033	Beach	0.124	5	2.86	1.596	.052	0.217	0–0.439	Neutral	0.32
	Inland water	0.006	0	0.14	0.141		0.000	0–0	Negative	−1
	Manzanillo forest	0.430	15	9.90	2.631		0.652	0.396–0.908	Neutral	0.43
	Dry‐forest	0.420	3	9.67	4.599		0.130	0–0.311	Negative	−0.66
	Road paved	0.019	0	0.43	0.431		0.000	0–0	Negative	−1
	Total	1	23	23	9.40					
LF0216	Inland water	0.018	0	0.57	0.566	.001	0.000	0–0	Negative	−1
	Manzanillo forest	0.428	4	13.28	6.483		0.129	0–0.279	Negative	−0.67
	Dry‐forest	0.511	27	15.85	7.850		0.871	0.721–1.021	Positive	0.73
	Road paved	0.042	0	1.31	1.309		0.000	0–0	Negative	−1
	Total	1	31	31	16.21					
LF1233	Beach	0.026	0	0.74	0.743	.152	0.000	0–0	Negative	−1
	Inland water	0.020	0	0.58	0.582		0.000	0–0	Negative	−1
	Manzanillo forest	0.327	5	9.48	2.120		0.172	0–0.353	Neutral	−0.40
	Dry‐forest	0.600	24	17.41	2.493		0.828	0.647–1.008	Positive	0.52
	Road paved	0.027	0	0.78	0.779		0.000	0–0	Negative	−1
	Total	1	29	29	6.72					
LF1234	Manzanillo forest	0.197	5	5.90	0.137	.255	0.167	0.004–0.33	Neutral	0
	Dry‐forest	0.781	23	23.42	0.008		0.767	0.582–0.952	Neutral	−0.04
	Road paved	0.023	2	0.68	2.589		0.067	0–0.176	Neutral	0.51
	Total	1	30	30	2.73					

## 4 DISCUSSION

### 4.1 Exploring Darwin's finch movement and space use

We have shown that radio tags can be used to track the movements of individual medium ground finches for at least a 3‐week period and, therefore, determine their habitat selection patterns. Other, mostly arboreal, finches, such as the woodpecker finch and the mangrove finch, have been tracked in previous studies (Cunninghame et al., 2013, 2015, 2017; Fessl et al., 2010), yet our study is the first to use VHF tracking for any ground finch species. The resulting fine‐scale temporal and spatial data on activity patterns revealed aspects of finch biology that are invaluable for understanding the ecology and evolution of these birds. For example, we identified nesting places, foraging areas, and roosting sites that together delimit the home range of these ground finches. All the nests were located on cacti, which are found in abundance in the dry‐forest (Grant, 1999). Furthermore, daily movement patterns of the finches from the arid habitat to the coastal habitat illustrate the importance of movement and multiple habitat use during the breeding season.

No estimates of the home range size of breeding *Geospiza fortis* are available in the literature for comparison, since previous studies focused on nesting territory (i.e., the confined area around the nest), estimated from observations of males’ territorial behavior (Boag & Grant, 1984). Using VHF tracking, we were able to follow the finches not only over their nesting territory but also over the entire area in which they live and move (i.e., the full home range for the given period of time). The smallest home range we estimated using the minimum convex polygon method was 33,700 m^2^ (3.37 ha, Figure 1a, Table 2), and the largest range was 172,500 m^2^ (17.25 ha, Figure 1b, Table 2) with an average of 101,600 m^2^ (10.16 ha ±2.25 ha *SE*, *N* = 5). The only previous estimates of nesting territory size for *G*.* fortis*, calculated as minimum convex polygons, are 0.2% (203.6 m^2^) and 0.5% (477.8 m^2^), respectively, of the estimated home range size in this study (Boag & Grant, 1984), indicating that relatively large areas are required to meet the spatial needs of breeding finches.

Home range size and habitat selection patterns often vary during the annual cycle (Rühmann et al., 2019; Stanley et al., 2021; Wiktander et al., 2001). We found that the smallest (7.58 ha) and the largest (29.09 ha) home range size corresponded to an egg‐laying bird (JP4645; Tables 1 and 2) and a chick‐rearing bird (KGSK2033), which is consistent with general expectations for birds (e.g., Kolts & McRae, 2017; Zurell et al., 2018). However, we also found marked differences between both nest‐building males (LF0216 and LF1233 with home ranges of 14.86 ha and 24.71 ha, respectively). With a small sample of 5 individuals that differed in sex and breeding stage, we are limited in the strength of inference that can be made about how these factors impact range sizes.

Our data also revealed the existence of roosting activity in the Manzanillo forest and mangroves close to the sea (ranging from 0 m to 800 m). Darwin's finches typically aggregate during the nonbreeding season to form large foraging flocks during the day (Schluter, 1984). Our observations indicate that they may display gregarious behavior also during the breeding season (except during incubation), even if roosting together at night requires birds to travel much longer distances than diurnal activities. This observation challenges the assumption that Darwin's finches roost in or close to their nests (e.g., <300 m; Boag & Grant, 1984) and suggests that communal roosting may be advantageous to finches in general and nonincubating individuals in particular, although the exact benefits of roosts (e.g., reduced predation risk, foraging efficiency, or thermoregulation costs (Beauchamp, 1999; Eiserer, 1984; Lack, 1968; Tebbich et al., 2010; Ward & Zahavi, 1973)) remain to be explored.

### 4.2 Data quantity and quality ascertainment

Rarefaction curves of the minimum convex polygon reached a plateau at approximately 30 location points, indicating that moderate tracking effort is required to accurately calculate the home range size of a finch during the breeding season (Figure A6). This minimum number of fixes is similar to that reported for other breeding birds (Bechtoldt & Stouffer, 2005; Camacho et al., 2014), although more locations would probably be required for home range size estimation outside the breeding season due to flocking, postfledging dispersal, or seasonal movements (Gula & Theuerkauf, 2013). Our data also suggest that radio‐tracking methods may be useful to collect enough data points even in the largely inaccessible landscape (i.e., dense vegetation, volcanic substrate) of the Galápagos (e.g., to infer the nest location based on diurnal activity locations; Figure A9). Most importantly, our data proved useful for shedding new light on key aspects of the natural history of Darwin's finches, such as their breeding behavior, nest location, commuting behavior, and habitat selection and use.

### 4.3 Identifying limitations of radio‐tracking in finches

It is important to note that this is a pilot study aimed at providing preliminary data to test the utility of radio‐tracking for improving understanding of movement ecology in Darwin's finches. Constraints on the duration of the tracking period due to the short (~3 weeks) battery life of miniature radio‐transmitters restricted the volume of data that could be collected. This is a common limitation in telemetry studies of small, fast‐moving birds, although its impact on home ranges and habitat selection estimates appears to be small compared to larger animals (Mitchell et al., 2019). In addition, we identified some challenges and limitations on telemetry specific to our study system. First, complex topography and dense vegetation in parts of the arid coastal zone represent a difficult environment to track finches. For example, we were unable to find the nest of the individual LF1234 (but see Figure A9). This limitation could be overcome using drones equipped with an antenna to track finches and with an onboard camera to film the location of the nest (Desrochers et al., 2018). A second limitation was the labor‐intensive task of tracking finches with portable antennas in variable, but generally harsh, climatic conditions. A potential solution could be the implementation of automated radio tracking, consisting of a system of antennas distributed across the landscape, thus scanning a broader area with less effort (e.g., Cellular Tracking Technologies (CTT), Bridge et al., 2011; Motus et al., 2017); or with an open source telemetry system (Gottwald et al., 2019). Such a network of antennas scattered in the landscape would be particularly useful for determining the movement patterns of nonbreeding finches flocking and moving long distances. Third, we observed tag removal by one individual (LF0126), which has also been identified as a limitation in other telemetry studies (Rechetelo et al., 2016). Of course, our tagging approach was temporary, with tag retention only required long enough to complete the study (in our case 3 weeks). Finally, as is usually the case in radio‐tracking studies, bearing error increased with distance of detection (between the observer and the radio transmitter), as well as with reduced orthogonality of bearings (Fuller et al., 2005). Here again, using drones could provide a solution by enabling access to terrain that is difficult for humans to traverse, thereby allowing shorter distance of detection and fully orthogonal bearings. From our experience, a drone used for mapping purposes in another study on the Galápagos islands seemed to be ignored by the finches (personal observations).

To summarize, although there are some constraints on the use of telemetry with Galápagos finches, we believe that all are surmountable and should not prevent researchers from studying the movement ecology of the finches at the individual level. We highly encourage the pursuit of this study and, for that reason, we outline below three long‐standing questions about space use in finches that could be addressed using telemetry data.

### 4.4 Unanswered questions

#### 4.4.1 What ecological factors influence finch's home range size and location?

Many factors can influence the space use of birds, such as food availability, habitat composition and configuration, population density, predator–prey interactions, human disturbance, topography, nesting site availability, climatic conditions, sex, age, social status, and flocking (Rolando, 2002). In finches, territory size can change due to interrelated processes, such as fluctuations in rainfall (Grant, 1999; Smith et al., 1978), food availability (Schluter, 1984), and population densities (Boag & Grant, 1984), although the effect of the spatial scale of environmental variation and movement remains to be examined. Obtaining accurate territory size (and home range) estimates at multiple spatial scales (e.g., from core to edge) should enable researchers to better understand the scale‐specific mechanisms that shape territorial behavior in these birds. However, postfledging movements could be tracked to better understand the dispersal ecology of Darwin's finches (Gabela, 2007; Grant & Grant, 1989). Using radio‐transmitters for tracking nonbreeding adults could also help determine how much flocking increases the chances of locating new food patches and when defending a patch of resources becomes more costly than searching for new patches (De León et al., 2014; Schluter, 1984). Furthermore, the Galápagos landscape is changing due to urbanization and agricultural intensification. Human‐induced changes in the availability of resources might change the abundance and movement patterns of finches in certain environments, for example, due to the introduction of fruits in agricultural areas (Swarth, 1934), although tracking studies are needed to assess the true impact of these changes.

#### 4.4.2 How does finch movement impact their ecological interactions with other taxa?

Movement is a key component shaping ecological interactions and coexistence of species (Jeltsch et al., 2013). For example, the cactus finch (*G*.* scandens*) is dominant over the medium ground finch (*G*.* fortis*) (Boag & Grant, 1984). Therefore, it is possible that, under certain social and ecological contexts (e.g., shortage of nest sites (Orr, 1945) or nest‐building material in human‐altered areas), some finches compete for breeding territories or adjust their social behavior and/or home range size and location to local conditions, such as food availability, population density, and predation risk (Grant, 1999; Kleindorfer et al., 2009). Home range size and overlap between species could be studied in relation to diet overlap to better understand the interspecific or intraspecific (with respect to the different beak morphotypes in *G*.* fortis*; (Beausoleil et al., 2019)) determination of their space use (Boag & Grant, 1984).

Movement patterns in Darwin's finches can also be the basis of plant–animal interactions, for instance when granivorous finches disperse the seeds of the plants they use to build their nests (Camacho et al., 2018). Another application of telemetry on the finches could be to better understand the movement of finches in relation to the colonization and distribution of plants in the landscape, therefore, shedding light on nonrandom seed dispersal by birds. Conservation biologists could benefit from this information as movement patterns of the finches could determine the spread of invasive plants (Buddenhagen & Jewell, 2006; Camacho et al., 2018; Soria, 2006).

Darwin's finches are becoming exposed to avian pathogens from other organisms such as domestic chickens (*Gallus gallus*) (Parker, 2018; Wikelski et al., 2004). Tracking the movement of the potential hosts within islands can bring information on the potential proximity of birds that are infected by introduced pathogens and further our understanding on the spread of diseases (Food & Agriculture Organization of the United Nations, 2007). Therefore, studies gathering movement ecology information on finches could yield information about transmission of emergent imported diseases on the Galápagos affecting avian biodiversity.

#### 4.4.3 What factors influence roosting behavior in finches?

It has been noted that dense patches of *Opuntia* cacti in Daphne Major's crater were used as night roosts even for male finches holding territories (Boag & Grant, 1984). However, our understanding of the roosting behavior of Darwin's finches is limited and not much is known about the intrinsic and extrinsic factors driving variation in social behaviors. Communal roosting is relatively common in flocking birds (Beauchamp, 1999; Eiserer, 1984), and our observations suggest this behavior is present in ground finches. Nevertheless, the extent to which roosting behavior changes depending on the season (dry and wet) and life stage (breeding vs. nonbreeding) remains unclear.

Furthermore, a series of questions emerges from our observations. For example, does the type of roosting sites used differ in comparison to diurnal home ranges (Jirinec et al., 2016)? What are the fitness consequences of selecting a specific roosting location or habitat (e.g., in relation to predation risk; Eiserer, 1984)? Are there physiological and energetic advantages of selecting communal versus solitary roosting sites? Do roosting sites in urban areas compared to natural environments differ in their characteristics? Is the communal roosting behavior practiced only in coastal areas? How important is predation risk as a driving force for the evolution of roosting behavior in insular ecosystems compared to continental ones (Eiserer, 1984; Lack, 1968)? Are roosting sites only used by *G*. *fortis* or shared with other species of finches? Is there a sex bias in roosting location?

The finches we tagged were roosting in the coastal zone of the island, which has a denser vegetation cover than the nesting sites. This could have implications regarding thermoregulation costs and predation rates. Depending on within or between species interactions (e.g., dominance or the use of aggressive behavior), there could be competition for higher quality positions within the roosting site, with outcomes determined by factors such as social structure (Mezquida et al., 2005; Smith et al., 2008).

To conclude, our study opens up new avenues of research to better understand the roosting behavior and the movement ecology of Darwin's finches within islands. These can help understand the evolutionary dynamics of populations and complement our understanding of the ecology of the finches. The presence of urban and agricultural areas also provides a fertile ground to deepen our understanding of the effect of human activity on birds’ behavior.

## AUTHOR CONTRIBUTIONS

**Marc‐Olivier Beausoleil:** Conceptualization (equal); Data curation (equal); Formal analysis (equal); Funding acquisition (lead); Investigation (equal); Methodology (equal); Project administration (lead); Resources (equal); Software (lead); Supervision (lead); Validation (lead); Visualization (equal); Writing – original draft (equal); Writing – review & editing (equal). **Carlos Camacho:** Conceptualization (equal); Data curation (equal); Formal analysis (equal); Funding acquisition (equal); Investigation (equal); Methodology (lead); Project administration (equal); Resources (lead); Software (equal); Supervision (equal); Validation (equal); Visualization (equal); Writing – original draft (equal); Writing – review & editing (equal). **Julio Rabadán‐González:** Conceptualization (equal); Data curation (equal); Formal analysis (equal); Investigation (equal); Methodology (equal); Resources (equal); Software (equal); Supervision (equal); Validation (equal); Visualization (equal); Writing – original draft (equal); Writing – review & editing (equal). **Kristen Lalla:** Data curation (supporting); Investigation (supporting); Methodology (supporting); Resources (supporting); Writing – original draft (supporting); Writing – review & editing (supporting). **Roxanne Richard:** Investigation (supporting); Project administration (supporting); Resources (supporting); Visualization (supporting); Writing – review & editing (supporting). **Paola Carrion‐Avilés:** Investigation (supporting); Writing – review & editing (supporting). **Andrew P. Hendry:** Funding acquisition (equal); Resources (equal); Supervision (equal); Validation (equal); Writing – original draft (equal); Writing – review & editing (equal). **Rowan D. H. Barrett:** Supervision (equal); Writing – review & editing (equal).

### OPEN RESEARCH BADGES

This article has been awarded Open Data, Open Materials Badges. All materials and data are publicly accessible via the Open Science Framework at https://doi.org/10.6084/m9.figshare.18312863.

## Data Availability

Data and scripts are available on Dryad at https://doi.org/10.5061/dryad.qbzkh18kc.

## APPENDIX A

All appendix analysis and figures were produced in R (R Core Team, 2021)

**TABLE A1:** Roost count data

Time	Lake side			Time	Shoreline side			Total
	Entering	Leaving	Difference		Entering	Leaving	Difference	Balance
17:40 – 17:56	25	6	19	17:40 – 17:56	41	8	33	52
17:56 – 18:03	40	1	39	17:56 – 18:03	49	6	43	82
18:03 – 18:13	100	4	96	18:03 – 18:13	140	5	225	320
18:13 – 18:22	82	1	81	18:13 – 18:22	158	4	154	235
18:22 – 18:30	20	5	15	18:22 – 18:30	57	3	54	69
Subtotal	267	17	250	Subtotal	445	26	419	669

**TABLE A2:** JP4645 (female) roosting behavior and clutch state

Date	Time	Point name	Roosting place	Clutch state	Presumed stage
2019‐02‐28	5:42	R17	Manzanillo forest	Unknown	Unknown
2019‐03‐01	5:39	R21	Manzanillo forest	Unknown	Laying eggs
2019‐03‐02	–	–	Unknown	Unknown	Laying eggs
2019‐03‐03	9:45	Nest4	Unknown	3 eggs or more	Laying eggs
2019‐03‐04	5:28	R29	Nest (*Opuntia*)	Unknown	Incubating

**TABLE A3:** Spatial interaction between the birds. Proportion (in percentage) of the 95% home range and 50% core area of one bird covered by the home range of another bird

Band	Home range						Core area					
	JP4645	KGSK2033	LF0216	LF1233	LF1234	Average	JP4645	KGSK2033	LF0216	LF1233	LF1234	Average
JP4645	–	65.85	38.94	75.62	68.03	62.11	–	0	0	61.01	0	15.25
KGSK2033	17.15	–	14.50	34.07	45.52	27.81	0	–	0	0	6.02	1.55
LF0216	19.85	28.39	–	77.15	41.24	41.66	0	0	–	43.42	0	10.86
LF1233	23.19	40.11	46.41	–	60.43	42.53	17.32	0	20.03	–	18.30	13.91
LF1234	19.47	50.03	23.15	56.40	–	37.26	0	5.98	0	11.44	–	4.36
**Average**	19.92	46.09	30.75	60.81	53.80		4.33	1.50	5.01	28.97	6.14	

Note

Read the table as a row bird ID is overlapped with a certain area given in proportion of the bird in a corresponding column.

**TABLE A4:** Average proportion of home range and core area and number of individual bird locations in each habitat type

Habitat type	Proportion of habitat in finches’ space use (%)^a^			Number of points in each habitat for each bird					Total
	Home range	Core area		JP4645	KGSK2033	LF0216	LF1233	LF1234	
Beach	6.93	2.37		1	5	0	0	0	6
Inland water	0.95	0		0	0	0	0	0	0
Manzanillo forest	35.54	33.67		1	15	4	5	5	30
Dry‐forest	55.20	61.80		28	3	27	24	23	105
Road paved	1.39	2.15		0	0	0	0	2	3
Total	100.01^b^	99.99^b^		30	23	31	29	30	143

^a^ Calculated from the habitat polygon divided by the union of all birds’ home ranges (total area of home ranges).

^b^ Rounding imprecision.
